# Depression Among Patients with Chronic Cluster Headaches

**DOI:** 10.7759/cureus.5912

**Published:** 2019-10-15

**Authors:** Laraib Jumani, Reema Kumari, Deepak Kataria, Vinesh Kumar, Syed Muhammad Usama, Aakash Chandnani, Ujala Zubair

**Affiliations:** 1 Internal Medicine, Pakistan Institute of Medical Sciences, Islamabad, PAK; 2 Internal Medicine, Chandka Medical College Hospital, Larkana, PAK; 3 Internal Medicine, Ghulam Mohammad Mahar Medical College, Sukkur, PAK; 4 Internal Medicine, Dow University of Health Sciences, Karachi, PAK; 5 Internal Medicine, Jinnah Sindh Medical University, Karachi, PAK; 6 Miscellaneous, Dow University of Health Sciences, Karachi, PAK

**Keywords:** cluster headaches, depression, headache syndromes, quality of life

## Abstract

Introduction: Chronic headaches account for a significant proportion of people leading a poor quality of life. Chronic cluster headaches can be defined as episodes of headache usually around the eye in the pattern of a cluster lasting 15-180 minutes each followed by multiple similar episodes occurring at a frequency of 1-8 times per day.

Method: This cross-sectional study was conducted in Jinnah Postgraduate Medical Center, Karachi. One hundred patients who were diagnosed cases of chronic cluster headaches were asked to fill the Beck Hopelessness Scale (BHS), Headache Impact Test (HIT), and Hospital Anxiety and Depression Scale (HADS).

Results: Of our study subjects, 57 were males and 43 were females. The mean HIT-6 score among these patients was found to be 60.5±7.67 (p-value = 0.04). The mean BHS score among these patients was found to be 13±6.87. The mean HADS reporting anxiety (HADS-A) was found to be 12.54 ± 5.65; whereas, the mean HADS reporting depression (HADS-D) was found to be 7.65 ± 4.65.

Conclusion: Patients with chronic cluster headaches have higher scores than the general population. There is an association between headache syndromes and depression which require further investigation.

## Introduction

Chronic headaches account for a large proportion of people having a poor quality of life. There are various headache syndromes; cluster headaches are among one of them. These are usually unilateral headaches and rarely bilateral. It occurs usually in the pattern of cluster. It can be broadly classified into two categories: episodic cluster headache which occurs in episodes and chronic cluster headaches which is much more debilitating [1].

The World Health Organization (WHO) has recognized chronic headache syndromes as among the ten most disabling conditions in the world [2]. Some females have described cluster headaches as being much more painful than giving birth to a child [3]. Studies have shown that chronic headaches have been associated with cognitive decline. This can be explained by the involvement of structures in chronic cluster headache which impair the thinking and decision-making capability of a person during the periods of attack [4-5].

## Materials and methods

This is a cross-sectional study conducted at the department of neurology, Jinnah Postgraduate Medical Center in Karachi, Pakistan. Patients diagnosed with chronic cluster headaches were contacted via telephone. Informed consent was taken before including them in the study. They were asked to fill a survey assessing demographic variables along with scales assessing depression, anxiety, and hopelessness. The international classification of headache disorders (ICHD-II) was used for the diagnosis of cluster headache. This classification defines chronic cluster headaches as episodes of headache usually around the eye in the pattern of a cluster lasting 15-180 minutes each followed by multiple similar episodes occurring at a frequency of 1-8 times per day [6]. Patients with co-morbid conditions such as diabetes, hypertension and any other conditions were excluded from the study. Patients with addictions such as smoking, alcoholism, and any other substance use were excluded from the study. This was done to exclude the effect of these co-morbid conditions on anxiety, depression, and hopelessness among subjects. A total of 100 patients were selected. The sampling technique applied was random sampling.

Participants were asked to fill the Beck Hopelessness Scale (BHS), Headache Impact Test (HIT), and Hospital Anxiety and Depression Scale (HADS). The HIT-6 is a six-item scale that is a reliable and valid tool for assessing the impact of headache on the cognitive and social well being of an individual. The score range is from 36-78. It has been proven to have good internal consistency along with good test and re-test reliability. It is easy to use for researchers and clinicians [7].

The HADS is a 14-item scale used by clinicians to assess the level of anxiety and depression among patients. Seven items from this scale are used for assessment of anxiety and seven items are used for depression. The scoring ranges from 0-21 [8].

BHS is a 20-item questionnaire designed to measure hopelessness. It can measure hopelessness by assessment of three components: feelings about future, loss of motivation, and expectations [9].

## Results

Our study comprised of 100 patients. Of them 57 were males and 43 were females. The age range was 22-52 years. All of these patients were diagnosed with cases of chronic cluster headaches. The mean HIT-6 score among these patients was found to be 60.5±7.67 (p value = 0.04). The mean BHS score among these patients was found to be 13±6.87. Table 1 shows the mean of various components of the BHS score among our study subjects. The mean HIT-6 score among patients was found to be 60.52 ± 9.28. Scores greater than 50 are considered to have a significant impact of headache on the patient’s well being. The mean HADS reporting anxiety (HADS-A) was found to be 12.54 ± 5.65; whereas, the mean HADS reporting depression (HADS-D) was found to be 7.65 ± 4.65.

**Table 1:** Response of patients against the Beck Hopelessness Scale (BHS)

COMPONENTS OF THE BECK HOPELESSNESS SCALE	MEAN	STANDARD DEVIATION
I look forward to the future with hope and enthusiasm	0.7	0.67
I might as well give up because there is nothing I can do about making things better for myself	0.8	0.20
When things are going badly I am helped by saying they cannot stay this way forever	0.87	0.564
I cant imagine what my life would be like after ten years	0.54	0.76
I have enough time to accomplish the things I want to do.	0.65	0.34
In the future I expect to succeed in what concerns me the most.	0.8	0.6
My future seems dark to me.	0.34	0.65
I happen to be particularly lucky and expect to get more things in life than the average person.	0.65	0.87
I just can’t get the breaks and there is no reason that I will get in the future.	0.35	0.78
My past experiences have prepared me well for the future.	0.45	0.89
All I can see ahead of me is unpleasantness rather than pleasantness.	0.54	0.45
I don’t expect to get what I really want	0.65	0.25
When I look ahead at the future I expect to be happier than I am now.	0.56	0.59
Things just don’t work out the way I want them to.	0.76	0.56
I have great faith in future.	0.43	0.57
I never get what I want so its foolish to want anything.	0.56	0.54
Its very unlikely that I will get any real satisfaction in the future.	0.9	0.52
The future seems vague and uncertain to me	0.63	0.76
I can look forward to more good times than bad times.	0.78	0.43
There is no point of really trying to get anything because I wont get it.	0.47	0.75

## Discussion

Torkamani et al. have described differences between various aspects of routine life of healthy controls and those with cluster headaches either episodic or chronic. His study reports a mean HIT-6 score among patients with chronic cluster headaches to be 64.60 ± 4.81, mean BHS score was found to be 11.67±5.7, mean HADS-A score was found to be 11.63 ± 3.70, and HADS-D score was found to be 9.75±3.54 [10]. In contrast to the study by Torkamani et al., our study reports mean HIT-6 score of 60 ± 7.67, mean BHS score of 13 ± 6.87, mean HADS-A score of 12.54 ± 5.65, and mean HADS-A score of 7.65 ± 4.65.

Some studies have reported that cluster headaches have a much more adverse effect on the quality of life as compared to migraine headaches [11]. Symptoms of depression, anxiety, and suicidal thoughts are more common in patients with chronic headaches as compared to healthy controls [12].

Choi et al. concluded in his study that people with cluster headaches end up with more burden related to employment because of being less efficient due to debilitating cluster headaches [13]. Muneer et al. conducted a study in which he assessed the frequency of primary headache syndrome among patients with major depressive disorder. His results demonstrate that around one-third of patients with depression have any of one underlying chronic headache syndromes [14]. There can be an underlying association between chronic headache syndromes and depression and further studies are required to evaluate it. Some studies have demonstrated that tension-type headaches and migraines have a much greater association with depression than any other chronic headache syndromes [15].

An Italian based study concluded that a third of patients with chronic headache syndromes have major depression. In this study, depression was assessed using the Mini International Neuropsychiatric Instrument (MINI) in secondary and tertiary care hospitals [16].

The limitations of this study include a small sample size. In a third world country such as Pakistan, due to the limited availability of health care services, the majority of patients with cluster headaches remain undiagnosed. Our study only targets the population receiving treatment for cluster headaches. There is a chance that because of the treatment received, the level of depression, anxiety, and, hopelessness among this population will be significantly less than that of the population which remains undiagnosed.

Further studies assessing the differences in the routine life of patients with cluster headaches are required.

## Conclusions

It can be concluded that there is an association between chronic headache syndromes, depression, anxiety, and hopelessness. Further investigations can strengthen the association. Multi-modal treatment involving psychologists and psychotherapists can improve the quality of life among patients with chronic cluster headaches.
